# COVID-19 Information-Seeking and Prevention Behaviors in Florida, April 2020

**DOI:** 10.5888/pcd18.200575

**Published:** 2021-02-25

**Authors:** Justine Gunderson, Dwayne Mitchell, Keshia Reid, Melissa Jordan

**Affiliations:** 1 Florida Department of Health, Public Health Research Unit, Division of Community Health Promotion, Tallahassee, Florida

## Abstract

**Introduction:**

Coronavirus disease 2019 (COVID-19) surveillance can be enhanced by collecting population-level data on individual prevention measures. We described the use of a state-based, population-level surveillance system on COVID-19 prevention and information-seeking behaviors in Florida during the first month of survey administration.

**Methods:**

Beginning in April 2020, respondents of the Florida Behavioral Risk Factor Surveillance System were asked a series of 8 questions about sources of COVID-19 information and prevention behaviors. We analyzed the prevalence of information-seeking and prevention behaviors among respondents who answered at least 1 of the 8 questions (N = 1,004) overall, by demographic characteristics, and by the presence of chronic conditions.

**Results:**

Most respondents reported engaging in prevention behaviors, including handwashing (98.2%), reducing or avoiding travel (96.6%), avoiding crowds and public events (96.5%), and keeping household members at home (87.5%); however, the prevalence of prevention behaviors varied significantly by age, sex, and education. The most frequently reported source of COVID-19 information was the Centers for Disease Control and Prevention’s website (40.8%) followed by the Florida Department of Health’s website (32.9%). We found significant differences in information sources across all demographic and chronic condition subgroups. A larger proportion of respondents with chronic conditions (vs without chronic conditions) reported consulting their personal doctor for COVID-19 information.

**Conclusion:**

Understanding the uptake and characteristics associated with individual prevention and information-seeking behaviors at the population level facilitates COVID-19 response efforts. The rapid implementation of COVID-19–related questions in the Florida BRFSS provides a useful model for other population-based surveillance systems.

**Summary**

**What is already known on this topic?**

In response to the COVID-19 pandemic, surveillance systems began monitoring trends in reported cases. Additional data on prevention and COVID-19 information-seeking behaviors are needed at the population level to inform disease response.

**What is added by this report?**

We described the addition of 8 questions about sources of COVID-19 information and prevention behaviors to the Florida Behavioral Risk Factor Surveillance System. We assessed the prevalence of information-seeking and prevention behaviors among respondents both overall and among demographic and chronic disease subgroups to identify differences in behavioral responses to the outbreak.

**What are the implications for public health practice?**

Our findings point to the need for public health officials to recognize potential differences in prevention behaviors when developing public health awareness campaigns and to communicate COVID-19 information using multiple channels. Other population-based health surveys might consider adding similar COVID-19–related questions to enhance surveillance efforts.

## Introduction

Coronavirus disease 2019 (COVID-19) is an infectious respiratory illness caused by severe acute respiratory syndrome coronavirus 2 (SARS-CoV-2). COVID-19 was first identified in mainland China in December 2019 and rapidly spread to other countries and territories, including the United States (1). By late May 2020, the number of reported cases in the United States approached 1.7 million and the number of deaths surpassed 100,000 (2). Trends in the number of reported cases, laboratory tests, hospitalizations, and deaths are continuously updated by national, state, and local surveillance systems to monitor transmission of COVID-19. Although these data are critical in forecasting disease incidence, they do not characterize population-level prevention and information-seeking behaviors. Effective management of infectious diseases largely depends on community members staying informed and engaging in everyday preventive actions, such as social distancing (3).

To enhance COVID-19 surveillance in Florida, the Florida Department of Health (DOH) used the Behavioral Risk Factor Surveillance System (BRFSS) to conduct a population-based assessment of COVID-19 information-seeking and prevention behaviors starting in April 2020. The inherently flexible nature of the BRFSS survey instrument allows states to quickly gather data on urgent public health issues, as evidenced by successes monitoring influenza-like illness during the 2009 H1N1 pandemic, vaccine shortages during the 2004–2005 influenza season, and mosquito-bite prevention behaviors during the 2016 Zika virus outbreak (4–6). The objective of this study was to describe the implementation and analysis of COVID-19 questions in the Florida BRFSS and summarize findings from the first month of survey administration.

## Methods

The BRFSS is a collaborative effort between the Centers for Disease Control and Prevention (CDC) and state health departments to administer annual random-digit–dialed telephone surveys to the noninstitutionalized adult population aged 18 or older in all 50 states, the District of Columbia, and US territories (7). This population-based surveillance system is a premier source of state and national data on health risk behaviors, chronic health conditions, unintentional injuries, and preventive health practices (7). BRFSS data are used to prioritize health issues, inform prevention programs, and identify populations at risk of chronic disease and disability. The Florida DOH has administered the BRFSS annually since 1986, with 16,959 Florida residents interviewed in 2019, the most recent complete year of survey administration.

Beginning in April 2020, Florida BRFSS respondents were asked a series of 8 questions about sources of COVID-19 information and prevention behaviors. All questions stemmed from the initial prompt, “As a result of COVID-19 concerns, are you doing any of the following?” Three questions asked about whether respondents consult 1) the CDC website 2), the Florida DOH website, and 3) their personal doctor for COVID-19 information. Five additional questions asked about whether respondents were 1) avoiding crowds and public events, 2) reducing or avoiding travel, 3) keeping household members at home while the outbreak lasts, 4) washing hands frequently, and 5) engaging in any other prevention activities. Each of the 8 questions was assessed by using dichotomous yes and no response options. Additionally, the question about any other prevention activities allowed for open-ended responses, resulting in a range of answers.

Data in this study are based on 1,004 respondents in the April 2020 sample (79.4%; n = 1,265) who answered at least 1 of the 8 COVID-19–related questions. We evaluated the prevalence of consulting each source of COVID-19 information and individual prevention behaviors overall and by demographic characteristics, including age group (18–44, 45–64, ≥65 ), sex (male, female), race/ethnicity (non-Hispanic White, non-Hispanic Black, Hispanic) education (<high school graduate, high school graduate/GED, some college or college graduate), annual household income (<$25,000, $25,000–$49,000, ≥$50,000), and marital status (married/unmarried couple, not married/not coupled). We also analyzed results by the presence of underlying chronic conditions that may increase the risk for severe illness from COVID-19, including diabetes, lung conditions (chronic obstructive pulmonary disease, emphysema, or chronic bronchitis), and heart conditions (angina or coronary heart disease) (8). The presence of chronic conditions was based on a positive response to the question “Have you ever been told by a doctor, nurse, or health professional that you have [CONDITION]?” In addition to examining differences in COVID-19 prevention behaviors and information sources by each chronic condition type, the number of these conditions (diabetes, chronic obstructive pulmonary disease, emphysema, chronic bronchitis, and angina or coronary heart disease) was summed and grouped into 3 mutually exclusive categories (0, 1, or ≥2 chronic conditions) for each respondent to create a combined chronic condition indicator that allowed us to examine differences by comorbidity status.

All numbers, percentages, and 95% CIs presented are unweighted. We used Rao–Scott χ^2^ tests to evaluate bivariate differences. We set significance at *P* < .05 for comparisons between sex, marital status, and individual chronic conditions in response to COVID-19 questions. To adjust for testing of multiple comparisons between age, race/ethnicity, education, income, and combined chronic condition indicator groups, we used a Bonferroni correction. Differences for multiple comparisons were significant if *P* < .017 (.05/3). We used SAS version 9.4 (SAS Institute, Inc) to perform all analyses.

## Results

Most respondents (45.6%) were aged 65 or older (Table 1). More than half (57.7%) were female. Most respondents were non-Hispanic White (79.7%), followed by Hispanic (12.8%), and non-Hispanic Black (7.5%). Nearly two-thirds (62.6%) had attended some college or were college graduates, and 39.3% reported a household income of $50,000 or more. Nearly 54% of respondents reported being married or coupled. More than one-fourth (26.0%) reported at least 1 chronic condition.

**Table 1:** Percentage Distribution of Characteristics of Sample (N = 1,004) and Responses to COVID-19 Questions, Behavioral Risk Factor Surveillance System, Florida, April 2020

Characteristic	No.	% (95% CI)
**Age, y**		
18–44	227	22.8 (20.2–25.5)
45–64	314	31.6 (28.7–34.5)
≥65	453	45.6 (42.5–48.7)
**Sex**		
Male	425	42.3 (39.2–45.3)
Female	580	57.7 (54.7–60.8)
**Race/ethnicity**		
Non-Hispanic White	760	79.7 (77.2–82.3)
Non-Hispanic Black	71	7.5 (5.8–9.1)
Hispanic	122	12.8 (10.7–14.9)
**Education**		
<High school graduate	96	9.6 (7.7–11.4)
High school graduate/GED	280	27.9 (25.1–30.6)
Some college or college graduate	629	62.6 (59.6–65.6)
**Annual household income, $**		
<25,000	279	33.5 (30.3–36.7)
25,000–49,000	227	27.3 (24.2–30.3)
≥50,000	327	39.3 (35.9–42.6)
**Marital status**		
Married/unmarried couple	537	53.6 (50.6–56.7)
Not married/not coupled	464	46.4 (43.3–49.4)
**Chronic conditions**		
Diabetes	145	14.4 (12.3–16.6)
Lung conditions^a^	129	12.9 (10.9–15.0)
Heart conditions^b^	78	7.8 (6.2–9.5)
No chronic conditions^c^	732	74.0 (71.3–76.8)
1 Chronic condition^c^	181	18.3 (15.9–20.7)
≥2 Chronic conditions^c^	76	7.7 (6.0–9.3)
**COVID-19 information sources**		
CDC website	410	40.8 (37.8–43.9)
Florida DOH website	330	32.9 (30.0–35.8)
Personal doctor	240	24.0 (21.3–26.6)
**Prevention behaviors**		
Avoiding crowds and public events	969	96.5 (95.4–97.7)
Reducing or avoiding travel	969	96.6 (95.5–97.7)
Keeping household members at home	862	87.5 (85.4–89.6)
Washing hands frequently	986	98.2 (97.4–99.0)
Other measures	634	63.3 (60.3–66.3)

Abbreviations: CDC, Centers for Disease Control and Prevention; COVID-19, coronavirus disease 2019; DOH, Department of Health.

a Lung conditions are chronic obstructive pulmonary disease, emphysema, or chronic bronchitis.

b Heart conditions are angina or coronary heart disease.

c Chronic conditions are diabetes, chronic obstructive pulmonary disease, emphysema, or chronic bronchitis; angina or coronary heart disease.

The most frequently reported source of COVID-19 information was the CDC website (40.8%), followed by the Florida DOH website (32.9%), and a personal doctor (24.0%) (Table 1). The most common COVID-19 prevention behavior was frequent handwashing, a precaution practiced by nearly all respondents (98.2%). Most respondents also reported reducing or avoiding travel (96.6%), avoiding crowds and public events (96.5%), and keeping household members at home (87.5%). Less than two-thirds (63.3%) reported engaging in any other prevention activities. Among those who reported other prevention activities, frequently described practices were variations of wearing a mask, using hand sanitizer, disinfecting and sanitizing surfaces, and maintaining social distance from others.

Reported sources of COVID-19 information differed significantly by demographic characteristics and the presence of chronic conditions (Table 2). Respondents aged 18 to 44 had a significantly higher prevalence of consulting the CDC website (58.1%) and the Florida DOH website (48.9%) compared with respondents 65 or older (26.3% and 18.0%, respectively), but they had a significantly lower prevalence of consulting their personal doctor (17.7% vs 28.8%). A significantly larger proportion of women (35.5%) than men (29.4%) reported consulting the Florida DOH website and a significantly larger proportion of Hispanic respondents (51.6%) than non-Hispanic White respondents (38.4%) reported consulting the CDC website. Respondents with some college education or a college degree had a significantly higher prevalence of consulting the CDC website than respondents with less than a high school education (45.9% vs 32.3%), but they had a significantly lower prevalence of consulting their personal doctor (23.2% vs 35.4%). Similarly, respondents with a household income of $50,000 or more had a significantly higher prevalence of consulting the CDC website than respondents making less than $25,000 (50.5% vs 36.2%), but they had a significantly lower prevalence of consulting their personal doctor (18.1% vs 29.5%). Married or coupled respondents had a significantly higher prevalence of consulting both the CDC and Florida DOH websites compared with their not married or not coupled counterparts (48.0% vs 32.6% and 36.4% vs 29.2%, respectively).

**Table 2:** COVID-19 Information Sources, by Demographic Characteristics and Chronic Conditions of Sample (N = 1,004), Behavioral Risk Factor Surveillance System, Florida, April 2020^a^

Characteristic	CDC Website		Florida DOH Website		Personal Doctor	
	No.	% (95% CI)	No.	% (95% CI)	No.	% (95% CI)
**Age, y**						
18–44	132	58.1^b^ (51.7–64.6)	111	48.9^b^ (42.4–55.4)	40	17.7^c^ (12.7–22.7)
45–64	157	50.2^b^ (44.6–55.7)	134	42.8^b^ (37.3–48.3)	67	21.4 (16.9–26.0)
≥65	119	26.3 (22.2–30.3)	81	18.0 (14.4–21.5)	130	28.8 (24.6–32.9)
**Sex**						
Male	161	37.9 (33.3–42.5)	125	29.4^d^ (25.1–33.8)	90	21.3 (17.4–25.2)
Female	249	43.0 (39.0–47.0)	205	35.5 (31.6–39.4)	150	25.9 (22.3–29.5)
**Race/ethnicity**						
Non-Hispanic White	292	38.4^e^ (35.0–41.9)	246	32.5 (29.2–35.8)	173	22.8 (19.8–25.8)
Non-Hispanic Black	31	44.3 (32.6–55.9)	27	38.0 (26.7–49.3)	22	31.0 (20.2–41.8)
Hispanic	63	51.6 (42.8–60.5)	42	34.4 (26.0–42.9)	30	24.8 (17.1–32.5)
**Education**						
<High school graduate	31	32.3^f^ (22.9–41.7)	27	28.1 (19.1–37.1)	34	35.4^g^^,^ h (25.8–45.0)
High school graduate/GED	91	32.5^i^ (27.0–38.0)	79	28.2 (22.9–33.5)	61	21.8 (16.9–26.6)
Some college or college graduate	288	45.9 (42.0–49.8)	224	35.8 (32.0–39.5)	145	23.2 (19.9–26.5)
**Annual household income, $**						
<25,000	101	36.2^j^ (30.5–41.9)	91	32.7 (27.2–38.2)	82	29.5^k^ (24.1–34.9)
25,000–49,000	90	39.8^l^ (33.4–46.2)	73	32.2 (26.1–38.2)	59	26.0 (20.3–31.7)
≥50,000	165	50.5 (45.0–55.9)	135	41.5 (36.2–46.9)	59	18.1 (13.9–22.3)
**Marital status**						
Married/unmarried couple	258	48.0^m^ (43.8–52.3)	195	36.4^n^ (32.4–40.5)	129	24.1 (20.5–27.7)
Not married/not coupled	151	32.6 (28.3–36.9)	135	29.2 (25.0–33.3)	111	24.0 (20.1–27.9)
**Chronic conditions**						
Diabetes	44	30.6^o^ (23.0–38.1)	39	26.9 (19.7–34.1)	59	40.7^p^ (32.7–48.7)
Lung conditions	45	34.9 (26.6–43.1)	38	29.5 (21.6–37.3)	47	36.4^q^ (28.1–44.8)
Heart conditions	25	32.1 (21.7–42.4)	21	27.3 (17.3–37.2)	26	33.3^r^ (22.9–43.8)
No chronic conditions	321	43.9^s^ (40.3–47.5)	258	35.3^t^ (31.9–38.8)	146	20.0^u^^,^ v (17.1–22.9)
1 Chronic condition	60	33.3 (26.4–40.2)	42	23.3 (17.1–29.5)	54	29.8 (23.2–36.5)
≥2 Chronic conditions	25	32.9 (22.3–43.5)	26	34.2 (23.5–44.9)	34	44.7 (33.5–55.9)

Abbreviations: CDC, Centers for Disease Control and Prevention; COVID-19, coronavirus disease 2019; DOH, Department of Health.

a Rao–Scott χ^2^ tests were used to determine *P* values. *P* < .05 was considered significant for sex (male vs female), marital status (married/couple vs not married/couple) and individual chronic condition comparisons (diabetes vs no diabetes, lung conditions vs no lung conditions, and heart conditions vs no heart conditions). The Bonferroni correction (*P* < .017) was applied when multiple comparisons were made between age, race/ethnicity, education, annual household income, and number of chronic conditions.

b *P* < .001 compared with respondents aged ≥65.

c *P* = .002 compared with respondents aged ≥65.

d *P* = .04 compared with female sex.

e *P* = .006 compared with Hispanic ethnicity.

f *P* = .01 compared with some college or college graduate.

g *P* = .008 compared with high school graduate/GED.

h *P* = .01 compared with some college or college graduate.

i *P* < .001 compared with some college or college graduate.

j *P* < .001 compared with annual household income ≥$50,000.

k *P* = .001 compared with annual household income ≥$50,000.

l *P* = .01 compared with annual household income ≥$50,000.

m *P* < .001 compared with not married/couple.

n *P* = .01 compared with not married/couple.

o *P* = .007 compared with respondents without diabetes (n = 366; 42.5% [95% CI, 39.2%–45.9%]).

p *P* < .001 compared with respondents without diabetes (n = 181; 21.1% [95% CI, 18.4%–23.9%]).

q *P* < .001 compared with respondents without lung conditions (n = 191; 22.1% [95% CI, 19.3%–24.8%]).

r *P* = .04 compared with respondents without heart conditions (n = 209; 22.8% [95% CI, 20.1%–25.6%]).

s *P* = .01 compared with respondents with 1 chronic condition.

t *P* = .002 compared with respondents with 1 chronic condition.

u *P* = .004 compared with respondents with 1 chronic condition.

v *P* < .001 compared with respondents with ≥2 chronic conditions.

A significantly larger proportion of respondents with diabetes reported consulting their personal doctor compared with respondents without diabetes (40.7% vs 21.1%). Likewise, a significantly larger proportion of respondents with lung conditions reported consulting their personal doctor compared with respondents without lung conditions (36.4% vs 22.1%). Respondents with 1 chronic condition or 2 or more chronic conditions had a significantly higher prevalence of consulting their personal doctor compared with respondents with no conditions (29.8% and 44.7% vs 20.0%, respectively). Compared with respondents with no conditions, respondents with 1 chronic condition were less likely to report consulting the CDC website (33.3% vs 43.9%) and the Florida DOH website (23.3% vs 35.3%) (Table 2).

The prevalence of engaging in COVID-19 prevention behaviors also differed significantly by demographic characteristics (Table 3). A significantly smaller proportion of respondents aged 65 or older reported keeping family members at home compared with those aged 18 to 44 (84.3% vs 91.1%). Although the prevalence of prevention behaviors was high among men and women, a significantly higher proportion of women than men reported avoiding crowds and public events (97.8% vs 94.8%), reducing or avoiding travel (98.3% vs 94.3%), frequent handwashing (99.1% vs 96.9%), and engaging in other prevention activities (67.0% vs 58.4%). We found several significant differences in prevention behaviors by education level. Compared with respondents with less than a high school diploma, respondents with some college or a college degree were more likely to report avoiding crowds and public events (97.8% vs 91.7%). Respondents with some college or a college degree were also more likely to report engaging in other prevention behaviors compared with respondents with less than a high school diploma or with a high school diploma/GED (68.0% vs 46.9% and 58.6%, respectively).We found no significant differences in prevention behaviors by race/ethnicity or the presence of chronic conditions.

**Table 3:** COVID-19 Prevention Behaviors, by Demographic Characteristics and Chronic Conditions, Behavioral Risk Factor Surveillance System, Florida, April 2020^a^

Characteristic	Avoid Crowds or Public Events		Reduce or Avoid Travel		Keep Family Members Home		Frequent Handwashing		Other Measures	
	No.	% (95% CI)	No.	% (95% CI)	No.	% (95% CI)	No.	% (95% CI)	No.	% (95% CI)
**Age, y**										
18–44	215	94.7 (91.8–97.6)	217	95.6 (92.9–98.3)	204	91.1^b^ (87.3–94.8)	223	98.2 (96.5–100.0)	133	58.6 (52.2–65.0)
45–64	306	97.5 (95.7–99.2)	299	95.5 (93.2–97.8)	279	89.7 (86.3–93.1)	306	97.8 (96.1–99.4)	211	67.4 (62.2–72.6)
≥65	437	96.7 (95.0–98.3)	443	98.0 (96.7–99.3)	370	84.3 (80.9–87.7)	446	98.5 (97.3–99.6)	285	63.3 (58.9–67.8)
**Sex**										
Male	402	94.8^c^ (92.7–96.9)	400	94.3^d^ (92.1–96.5)	362	86.4 (83.1–89.7)	412	96.9^e^ (95.3–98.6)	248	58.4^f^ (53.7–63.0)
Female	567	97.8 (96.6–99.0)	569	98.3 (97.2–99.3)	500	88.3 (85.7–91.0)	574	99.1 (98.4–99.9)	386	67.0 (63.2–70.9)
**Race/ethnicity**										
Non-Hispanic White	736	97.0 (95.7–98.2)	733	96.7 (95.4–98.0)	645	86.5 (84.0–88.9)	743	97.9 (96.9–98.9)	491	64.9 (61.5–68.3)
Non-Hispanic Black	69	97.2 (93.3–100.0)	68	95.8 (91.1–100.0)	62	88.6 (81.1–96.0)	71	100.0 (100.0–100.0)	37	52.9 (41.1–64.6)
Hispanic	114	93.4 (89.0–97.8)	118	96.7 (93.6–99.9)	111	93.3 (88.8–97.8)	121	99.2 (97.6–100.0)	77	63.1 (54.5–71.7)
**Education**										
<High school graduate	88	91.7^g^ (86.1–97.2)	92	95.8 (91.8–99.8)	81	85.3 (78.1–92.4)	93	96.9 (93.4–100.0)	45	46.9^h^ (36.9–56.9)
High school graduate/GED	267	95.4 (92.9–97.8)	270	96.4 (94.3–98.6)	242	88.6 (84.9–92.4)	276	98.6 (97.2–100.0)	164	58.6^i^ (52.8–64.4)
Some college or college graduate	614	97.8 (96.6–98.9)	607	96.8 (95.4–98.2)	539	87.4 (84.7–90.0)	617	98.2 (97.2–99.3)	425	68.0 (64.3–71.7)
**Annual household income, $**										
<25,000	264	94.6 (92.0–97.3)	267	96.0 (93.7–98.3)	235	85.5 (81.3–89.6)	275	98.6 (97.2–100.0)	172	61.6 (55.9–67.4)
25,000–49,000	216	95.2 (92.4–98.0)	219	96.5 (94.1–98.9)	196	87.5 (83.2–91.8)	220	96.9 (94.7–99.2)	146	64.6 (58.4–70.8)
≥50,000	320	98.2 (96.7–99.6)	314	96.0 (93.9–98.1)	293	90.7 (87.5–93.9)	323	98.8 (97.6–100.0)	220	67.3 (62.2–72.4)
**Marital status**										
Married/unmarried couple	522	97.2 (95.8–98.6)	522	97.4 (96.0–98.7)	488	91.6 (89.2–93.9)	527	98.1 (97.0–99.3)	359	67.0 (63.0–71.0)
Not married/not coupled	444	95.9 (94.1–97.7)	443	95.7 (93.8–97.5)	370	82.6 (79.1–86.1)	455	98.3 (97.1–99.5)	272	59.0 (54.5–63.5)
**Chronic conditions**										
Diabetes	140	96.6 (93.6–99.5)	141	97.2 (94.6–99.9)	122	85.9 (80.2–91.6)	142	97.9 (95.6–100.0)	86	59.3 (51.3–67.3)
Lung conditions	126	97.7 (95.1–100.0)	124	96.1 (92.8–99.5)	114	89.1 (83.6–94.5)	126	97.7 (95.1–100.0)	87	68.5 (60.4–76.6)
Heart conditions	76	97.4 (93.9–100.0)	72	93.5 (88.0–99.0)	65	84.4 (76.3–92.5)	77	98.7 (96.2–100.0)	50	64.1 (53.4–74.8)
No chronic conditions	706	96.6 (95.3–97.9)	708	96.9 (95.3–97.9)	634	88.4 (86.1–90.8)	718	98.2 (97.3–99.2)	468	64.1 (60.6–67.6)
1 Chronic condition	174	96.1 (93.3–98.9)	171	95.0 (91.8–98.2)	146	83.0 (77.4–88.5)	177	97.8 (95.6–99.9)	102	57.0 (49.7–64.2)
≥2 Chronic conditions	75	98.7 (96.1–100.0)	74	97.4 (93.8–100.0)	68	89.5 (82.6–96.4)	75	98.7 (96.1–100.0)	54	71.1 (60.8–81.3)

Abbreviation: COVID-19, coronavirus disease 2019.

a Rao–Scott χ^2^ tests were used to determine *P* values. *P* < .05 was considered significant for sex (male vs female), marital status (married/couple vs not married/couple) and individual chronic condition comparisons (diabetes vs no diabetes, lung conditions vs no lung conditions, and heart conditions vs no heart conditions). The Bonferroni correction (*P* < .017) was applied when multiple comparisons were made between age, race/ethnicity, education, annual household income, and number of chronic conditions.

b *P* = .015 compared with respondents aged ≥65.

c *P* = .01 compared with female sex.

d *P* < .001 compared with female sex.

e *P* = .01 compared with female sex.

f *P* = .005 compared with female sex.

g *P* = .001 compared with some college/college graduate.

h *P* < .001 compared with some college/college graduate.

i *P* < .006 compared with some college/college graduate.

## Discussion

On April 1, 2020, Florida had approximately 7,000 confirmed cases of COVID-19 among its residents. By the end of April, this number was approaching 33,000 (9). Monitoring the adoption of prevention behaviors and engagement with information sources in the community as cases increase is critical in aiding COVID-19 mitigation strategies. To our knowledge, our study is one of the first to describe the use of a population-based surveillance system to rapidly gather data on COVID-19 information-seeking and prevention behaviors. Our results provide a timely assessment of these behaviors during the early stages of the outbreak. Encouragingly, most of respondents engaged in all 4 prevention behaviors: frequent handwashing, reducing or avoiding travel, avoiding crowds and public events, and keeping household members at home. Further, levels of engagement with information sources were high. More than 1 in 3 survey respondents reported consulting the CDC website and nearly one-third reported consulting the Florida DOH website. We found several significant differences in information-seeking and prevention behaviors by demographic characteristics and the presence of chronic conditions. For example, a significantly lower proportion of men than women in our sample reported avoiding crowds, reducing travel, and frequent handwashing, mirroring the results of recent research examining differences by sex in COVID-19 prevention behaviors (10). Despite their greater risk for COVID-19 complications, respondents with chronic conditions did not differ significantly from respondents without chronic conditions in engagement of prevention behaviors. However, a significantly larger proportion of respondents with chronic conditions, compared with respondents with no chronic conditions, reported consulting their personal doctor for COVID-19 information. Our findings emphasize the importance of recognizing potential differences in prevention behaviors when developing public health awareness campaigns. Our findings also illustrate the need for COVID-19 information to be conveyed through multiple channels, including during patient–physician interactions, to further enhance health literacy among populations at high risk for severe illness, such as those with chronic conditions.

Our study has several limitations. First, to quickly begin gathering information on prevention and information-seeking behaviors, our analysis focused on data collected during the first month of implementation of COVID-19 questions on the Florida BRFSS survey. Data from the BRFSS is typically combined over 12 months or multiple years and weighted to create a sample that represents the overall population. Estimates based on 1 month of unweighted data may not represent the overall population of Florida. However, these estimates provide initial insight into behaviors that took place in the state during the early stages of the outbreak. Second, time constraints inherent in quickly gathering data limit the ability to conduct in-depth cognitive testing and pretesting or examine the validity of survey questions. As data collection progresses, or information needs change, efforts will be made to ensure questions generate the type of data needed. Third, BRFSS data do not represent people who do not have access to telephones. Fourth, all responses are self-reported and may be susceptible to social desirability bias, resulting in potential overreporting of prevention behaviors. Despite these limitations, harnessing the flexible nature of the BRFSS allowed for the rapid assessment of prevention and information-seeking behaviors among residents of the third most populous state during the early days of the pandemic, when information was particularly critical. Furthermore, although our findings cannot be generalized to other locations in the United States, our use of the Florida BRFSS to assess COVID-19 information sources and prevention behaviors may provide a valuable template for other population-based health surveillance systems that may be considering collecting similar information.

The time-sensitive addition of questions on COVID-19–related concerns to the Florida BRFSS expands disease surveillance efforts to capture information on individual prevention and information-seeking behaviors in the community setting. Our findings support COVID-19 response efforts across the state by providing a detailed picture of engagement with information sources and prevention behaviors both overall and among numerous demographic and chronic disease subgroups. To further strengthen data collection efforts and inform localized public health strategies, other states and territories might consider using the BRFSS to track COVID-19–related indicators as the pandemic unfolds. BRFSS administration is ongoing in Florida, and we will continue to monitor trends in information sources and prevention behaviors among residents and build on these initial findings as the outbreak evolves.
